# Prolonged Survival of a Refractory Acute Myeloid Leukemia Patient after a Third Hematopoietic Stem Cell Transplantation with Umbilical Cord Blood following a Second Relapse

**DOI:** 10.1155/2014/918708

**Published:** 2014-02-09

**Authors:** Suk-young Lee, Naoki Kurita, Koichiro Maie, Masanori Seki, Yasuhisa Yokoyama, Kazumi Suzukawa, Yuichi Hasegawa, Shigeru Chiba

**Affiliations:** Department of Hematology, Faculty of Medicine, University of Tsukuba, Tennodai 1-1-1, Tsukuba, Ibaraki 305-8575, Japan

## Abstract

Although hematopoietic stem cell transplantation (HSCT) has been considered to be the only way for potential cure of relapsed acute myeloid leukemia (AML), there has been no report on a third HSCT in patients with multiple relapsed AML. Here, we report a case of 53-year-old female who received a successful third allogeneic HSCT after relapse of AML following a second allogeneic HSCT. She was treated with a toxicity reduced conditioning regimen and received direct intrabone cord blood transplantation (CBT) using a single unit of 5/6 HLA-matched cord blood as a graft source. Graft-versus-host disease prophylaxis was performed with a single agent of tacrolimus to increase graft-versus-leukemia effect. She is in remission for 8 months since the direct intrabone CBT. This report highlights not only the importance of individually adjusted approach but also the need for further investigation on the role of HSCT as a treatment modality in patients with refractory or multiple relapsed AML.

## 1. Introduction

Decisions about how to treat patients with multiple relapsed AML are often based on the individual condition of each patient at relapse because no consensus on treatment of such patients has been established. Although there is no available clinical data as references in performing the third allogeneic HSCT currently, this has been performed sporadically at experienced transplant centers as one of options for treatment of patients with multiple relapsed AML, because the HSCT is regarded as the best way to achieve potential cure for refractory or relapsed AML. Here, we report a case who received the third allogeneic HSCT successfully without life-threatening complications and maintaining complete remission (CR) for 8 months.

## 2. Case Report

A female patient had been diagnosed with myelodysplastic syndrome, refractory anemia with excess blasts-2 (MDS, RAEB-2), at the age of 49. The disease progressed to AML showing nearly 50% blasts in bone marrow (BM), when she received bone marrow transplantation (BMT) from an HLA-matched unrelated donor. Her conditioning regimen comprised 120 mg/kg cyclophosphamide and 12 Gy that was administered as six doses of fractionated total body irradiation. Tacrolimus and short-term methotrexate were used for graft-versus-host disease (GVHD) prophylaxis. Engraftment was achieved on posttransplant day 15, and a BM examination on day 27 showed >99% donor chimerism. The patient developed grade I acute GVHD (skin, stage 1).

Relapse of AML was diagnosed 14 months after the transplantation. A combination chemotherapy comprising idarubicin and Ara-C was ineffective, and 30% leukemic blasts remained in the BM. A second HSCT was planned with cord blood as the graft source, because disease progression implied a need for an urgent care. A unit of 5/6 HLA-matched cord blood was transplanted after treatment with a preparation regimen comprising busulfan (12.8 mg/kg, i.v.) and cyclophosphamide (120 mg/kg) at 16 months from the first transplantation. Mycophenolate mofetil and tacrolimus were used for GVHD prophylaxis. Engraftment was achieved on posttransplant day 17. On day 35, remission was achieved in the BM. The patient had grade II acute GVHD (skin, stage 3; gut, stage 1) and a limited type of chronic GVHD on the skin.

**Figure 1:**
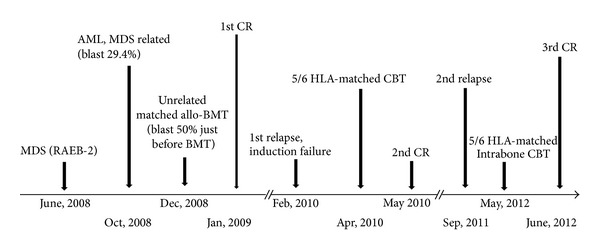
Time course of disease related to the HSCT. MDS, myelodysplastic syndrome; RAEB-2, refractory anemia with excess blast-2; allo-BMT, allogeneic bone marrow transplantation; CR, complete remission.

The patient experienced a relapse of AML 17 months after the CBT and showed 20% leukemic blasts in the BM. The same karyotype as that in the initial AML was observed. A third HSCT using cord blood as the graft source was planned in non-CR status after treatment with a hypomethylating agent, azacitidine, [1, 2] at the age of 53 again, because the patient had no significant comorbidities. A unit of 5/6 HLA-matched cord blood was transplanted with a preparative regimen comprising fludarabine (30 mg/m^2^/d for 5 days); ranimustine (MCNU) (150 mg/m^2^/d for 2 days); melphalan (140 mg/m^2^/d for 1 day) [3, 4]. Direct intrabone transplantation of a single unit of cord blood was performed [5, 6], with a total of 1 × 10^5^/kg CD34-positive cells and 3.2 × 10^7^/kg nucleated cells administered 25 months after the second HSCT. A single immunosuppressive agent, tacrolimus, was used for GVHD prophylaxis to expect a graft-versus-leukemia (GVL) effect. Engraftment was achieved on posttransplant day 14, and BM remission was confirmed on day 28. Acute skin stage 3 GVHD improved with steroid ointment treatment. BM examination 8 months after the direct intrabone CBT showed no evidence of leukemia recurrence (Figure 1).

## 3. Discussion

Here, we presented a case who successfully received a third allogeneic HSCT in her midfifties. Without any evidence from clinical trials, such decisions are made on a case-by-case basis and in accordance with an agreement between the transplant physicians in charge and the patient. Factors likely to have contributed to this case include early engraftment, minimum regimen-related toxicity (RRT), absence of serious infections, and mild GVHD that might have been associated with the GVL effect.

Although the decision of choosing an appropriate therapy is dependent on disease-related factors at relapse, HCST is known to be the only way to potentially cure relapsed AML. We presented an AML patient who achieved CR after the third allogeneic HSCT and maintained it for 8 months. Despite a second relapse, poor cytogenetics (monosomy 17), and relatively old age, the reason for reduced RRT and lasting relapse-free survival in this patient could likely be attributed to several factors. The most important factor is believed to be a prolonged relapse-free period after the previous HSCT. CR duration has been recognized as the most dominant factor affecting outcomes of salvage therapy in relapsed AML [7, 8]. In addition, the GVL effect is believed to have contributed to the extended period of relapse-free survival. It has been reported that CBT is beneficial in terms of reducing the frequency of GVHD while maintaining its GVL effect [9–12]. Furthermore, a study that presented the results of intrabone CBT showed an even lower frequency of GVHD with unchanged GVL effects [5]. In addition to these advantages, the dose of immunosuppressant was reduced early after transplantation, and only a single agent was used to increase the GVL effect in this patient. However, a longer follow-up period is required to prove the GVL effect, and the results of a large-scale prospective clinical trial should be waited to see the effect of intrabone CBT on the overall survival of AML patients. Direct intrabone CBT has also been reported as one way to overcome graft failure, one of the most serious complications of CBT in adult patients [5, 6]. Relatively rapid engraftment with cord blood was observed in this case, similar to that previously reported [5], and this was believed to be one reason for reduced transplantation-related complications. The reduced toxicity conditioning regimen, which has been reported to induce high antileukemic activity in patients with active and advanced AML with reduced RRT [3, 4], is also considered to contribute to reduced complications.

In conclusion, we described a case with relapsed AML who received HSCT for the third time as a curative treatment. This report has important implications, because to the best of our knowledge, this is the first report of a third HSCT in an AML patient, although it might be difficult to conclude that HSCT is a feasible treatment for most patients with refractory or relapsed AML based on only a single case. This report also highlights the need for further studies on the outcomes and prognosis-predicting factors in performing HSCT in multiple relapsed AML patients to reach a treatment consensus.
